# Prophylactic antibiotic treatment is superior to therapy on-demand in experimental necrotising pancreatitis

**DOI:** 10.1186/cc7118

**Published:** 2008-11-16

**Authors:** Stefan Fritz, Werner Hartwig, Ronny Lehmann, Katja Will-Schweiger, Mechthild Kommerell, Thilo Hackert, Lutz Schneider, Markus W Büchler, Jens Werner

**Affiliations:** 1 Department of General and Visceral Surgery, University of Heidelberg, Im Neuenheimer Feld 110, 69120 Heidelberg, Germany; 2 Section Infectiology, Institute of Hygiene, University of Heidelberg, Im Neuenheimer Feld 324, 69120 Heidelberg, Germany

## Abstract

**Introduction:**

High morbidity and mortality rates in patients with severe acute pancreatitis are mainly caused by bacterial superinfection of pancreatic necrosis and subsequent sepsis. The benefit of early prophylactic antibiotics remains controversial because clinical studies performed to date were statistically underpowered. Thus, the aim of this study was to evaluate on-demand versus prophylactic antibiotic treatment in a standardised experimental model.

**Methods:**

Treatment groups received meropenem either therapeutically 24 hours after induction of necrotising pancreatitis or prophylactically before development of pancreatic superinfection. At 24 and 72 hours, pancreatic injury was investigated by histology and translocation by bacterial cultures of pancreatic tissue and mesenteric lymph nodes. Septic complications were evaluated by blood cultures and survival.

**Results:**

Without antibiotic treatment, pancreatic superinfection was observed in almost all cases after induction of necrotising pancreatitis. The 72-hour-mortality rate was 42.9% and bacterial infection of mesenteric lymph nodes and bacteraemia was found in 87.5% of the surviving animals. Therapeutic administration of meropenem on-demand reduced bacteraemia to 50% and mortality to 27.3%. However, prophylactic antibiotic treatment significantly reduced bacteraemia to 25.0% (p = 0.04) and pancreatic superinfection as well as mortality to 0% (p < 0.001 and p = 0.05, respectively) compared with controls.

**Conclusions:**

In the present study both prophylactic and delayed antibiotic treatment on-demand reduced septic complications in a standardised setting of experimental necrotising pancreatitis. However, pancreatic superinfection, bacteraemia and mortality rates were reduced significantly by early treatment. Thus, in the absence of statistically relevant and well-designed clinical trials, the study demonstrates that prophylactic antibiotic treatment is superior to antibiotic treatment on-demand.

## Introduction

Although, the clinical course of acute pancreatitis is often mild and self-limiting, in 15% to 20% of patients severe necrotising pancreatitis develops, associated with local or systemic complications and high mortality rates [1]. One of the main reasons for fatal aggravation of the disease is bacterial superinfection of necrotic pancreatic tissue and the subsequent development of septic complications [2-4]. The risk of pancreatic superinfection is dependent on the amount of necrosis. The risk is about 20% if necrosis is less than 50% and increases up to 70% when pancreatic necrosis exceeds 50% [5].

In order to evaluate the benefit of prophylactic antibiotic application, a number of randomised controlled clinical trials have been published over the past 15 years [6-14]. Since the results were conflicting and most studies were of low methodological quality and/or statistically underpowered, meta-analyses have been performed to assess this important issue [3,15-17]. However, the results of several recent meta-analyses were also controversial. Some reported a benefit of prophylactic antibiotic treatment to avoid pancreatic superinfection [15-17], some did not find significant differences [18,19]. Others found that antibiotic prophylaxis was associated with decreased mortality but not with a decrease of extrapancreatic infections, infected pancreatic necrosis or operative treatment rates [3]. Due to these previously conflicting results, the Cochrane review in 2006 concluded that more trials were needed to confirm the benefits of antibiotic prophylaxis [3]. Consequently, until today there remain diverse treatment recommendations. Although most national and international guidelines for the management of acute pancreatitis recommend the use of early prophylactic broad-spectrum antibiotics [20,21], others do not [22,23].

There are a number of reasons why clinical trials and meta-analyses concerning the benefit of antibiotic prophylaxis in acute pancreatitis remain controversial. First, the severity of acute pancreatitis of patients included in studies showed great variations. For example, due to a lack of patient recruitment, several clinical trials included patients with an overall rate of pancreatic infected necrosis of 20% or less [6,7,9,10], which is lower than the expected 40% to 70% in the natural course of this disease as described by Beger and colleagues [24]. Furthermore, there was a great heterogeneity in management of acute pancreatitis. In some studies [14] patients were included up to 120 hours after onset of symptoms, at points in time when pancreatic necrosis was completely developed and in some cases already superinfected [24].

The most significant change in the clinical course of acute pancreatitis over the past decades has been the decrease in mortality from 40% to about 20%, mainly due to improvement of intensive care management. This decrease of mortality not only makes it difficult to compare recent studies to trials performed several years ago, it also has to be taken into account for calculations of trial sample size when considering mortality as an end-point. Also, many patients in recent studies showed relatively mild necrotising pancreatitis with less than 30% necrosis. Therefore, power calculations should be based on a pancreatic superinfection rate of about 20% compared with historical data, in which the rate of pancreatic necrosis was 50%. Theoretically, in order to prove that a reduction in infected necrosis decreases mortality from 20% to 10%, more than 3000 patients would have to be included in a clinical study [25]. In contrast to this, almost all trials performed in the past recruited only a small number of patients and even in the most recent published study, which was performed in 32 centres, the calculated number of patients (n = 240) was not reached and the study terminated early [14]. Therefore, all studies performed so far were statistically underpowered and it becomes clear that given the heterogeneity in patient management, it is difficult, or near impossible, to achieve the required number of patients in the future.

Due to variations in methodological quality, treatment regimens and the difficulty of including enough patients for a powerful study, the question of efficiency of antibiotic prophylaxis in acute necrotising pancreatitis can not be answered by clinical studies. In contrast to most medical treatments, where results from animal experiments are validated by clinical studies, in this case it was necessary to revert to animal experiments to create standardised settings in order to compare different antibiotic regimens. Thus, the aim of the present study was to evaluate the effects of antibiotic treatment in acute necrotising pancreatitis in a standardised animal model, and to investigate if there is a difference between the efficiency of early prophylactic antibiotic treatment and on-demand therapy after occurrence of proven infected necrosis.

## Materials and methods

### Experimental animals

Inbred male Wistar rats weighing 300 to 340 g (n = 68) were used for the experiments. Care was provided in accordance with the German law for use of laboratory animals (BGB1.I S. 1319). The study was approved by the Committee of Animal Care of the Regierungspräsidium Karlsruhe, Germany. Animals were allowed free access to food and water before starting the experiments.

### Anaesthesia and catheter placement

Surgical anaesthesia was induced by a short carbon dioxide narcosis followed by 40 mg/kg intramuscular ketamine (Ketanest^® ^S 25 mg/ml, Parke-Davis, Berlin, Germany) and 6 mg/kg xylazin (20 mg/ml Rompun^® ^2%, Bayer AG, Leverkusen, Germany). The left internal jugular vein was cannulated using a soft polyethylene catheter (0.5 mm ID, 0.8 mm OD, Braun Melsungen, Germany) for infusion regimens. The catheter was tunnelled subcutaneously to the suprascapular area and brought out through a steel tether that allowed free movement of the animals.

### Induction of acute necrotising pancreatitis

A detailed description of the induction technique has been reported previously [26]. In brief, the common bile duct was punctured and glycodeoxycholic acid (GDOC, Sigma-Aldrich Inc., St. Louis, USA) in glycyl-glycine-NaOH buffered solution (pH 8.0, room temperature) at a concentration of 10 mmol/L was infused in a time- (10 minute), pressure- (30 mmHg) and volume-controlled fashion (1.2 ml/kg). Subsequently, animals received a continuous intravenous infusion of 5 μg/kg/hour (6 ml/kg/hour) caerulein (Caerulein, Sigma-Aldrich Inc., St. Louis, USA) over six hours followed by volume substitution with 6 ml/kg/hour sodium chloride (NaCl) solution.

### Experimental design

**Figure 1:**
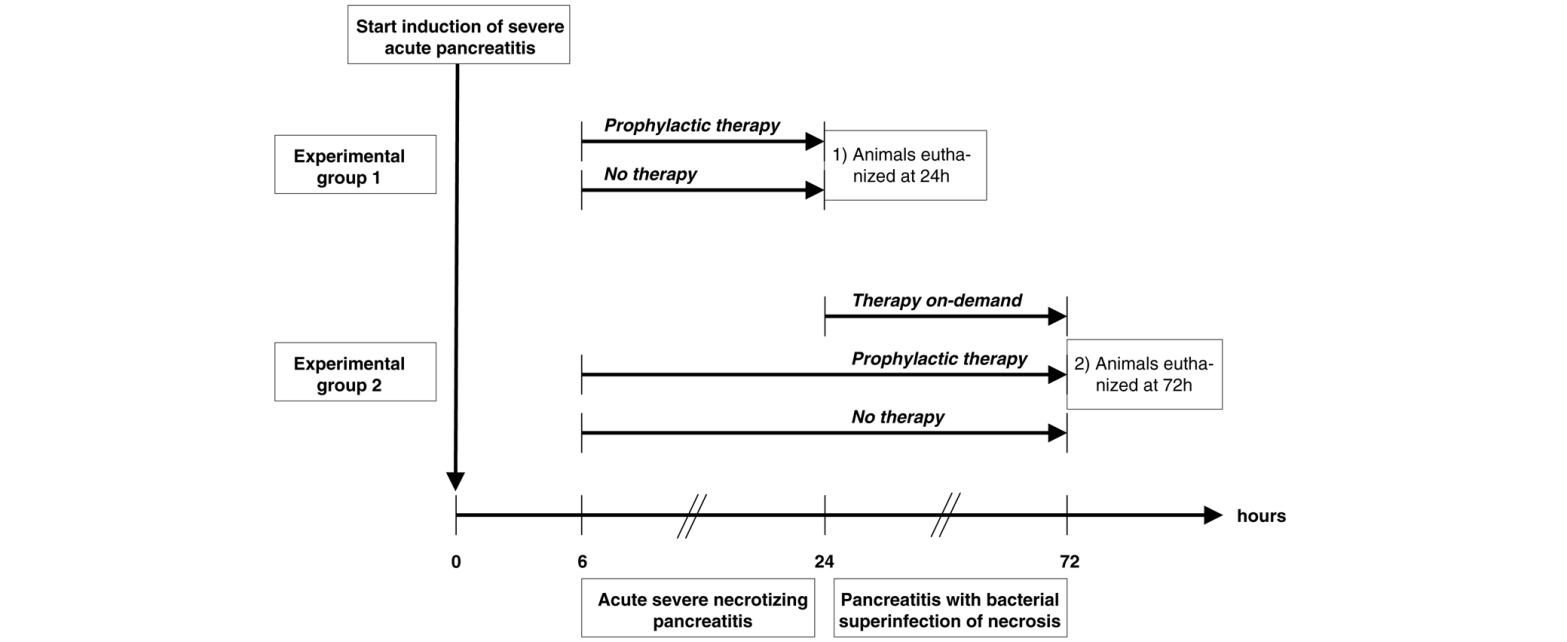
**Experimental design**. Animals in all treatment groups received induction of acute necrotising pancreatitis at time point 0. In experimental group 1, animals were euthanased at 24 hours. Prophylactic antibiotic treatment (starting six hours after induction of pancreatitis) was evaluated versus no antibiotic treatment. In a second experimental group, animal were euthanased at 72 hours. Meropenem therapy on-demand (starting at 24 hours after induction of pancreatitis) was compared with prophylactic treatment and controls.

In our first set of experiments, all animals were euthanased at 24 hours after induction of acute necrotising pancreatitis. At the end of the 24-hour period, pancreatic injury was evaluated by histological assessment of oedema, inflammation and necrosis. Animals (n = 6) receiving prophylactic antibiotic treatment (40 mg/kg intravenous Meropenem^®^, AstraZeneca GmbH, Wedel, Germany) every eight hours as a single dose started six hours after induction of acute pancreatitis) were compared with a control group (n = 6) that did not receive antibiotic treatment (Figure 1). Septic complications were evaluated by bacteriological assessments of blood, ascites, pancreas and mesenteric lymph nodes of both the colon and small bowel, as well as by 24-hour mortality rates.

In a second set of experiments, treatment effects of meropenem were evaluated 72 hours after induction of necrotising pancreatitis. Only animals that survived the first 24 hours after induction of acute pancreatic were included in the analysis. The results of our first experimental group demonstrated that superinfection of pancreatic necrosis is present in all animals at 24 hours. In order to imitate the clinical setting of severe necrotising pancreatitis, animals were treated with meropenem either prophylactically before infection of pancreatic necrosis was observed, or therapeutically after superinfection of pancreatic necrosis was present (Figure 1).

Thus, the first group of animals (n = 8) was infused with meropenem therapeutically after a therapy-free interval of 24 hours, which corresponds to treatment on-demand in the clinical setting. The second group (n = 8) received meropenem at six hours after induction of acute pancreatitis. At this point, pancreatic necrosis had already been established [26], but necrotic tissue was not yet superinfected because bacterial translocation occurs about 18 hours after induction of acute pancreatitis in severe experimental pancreatitis [27]. In the clinical setting this corresponds to a patient who presents to the hospital within the first three days after onset of the disease and receives antibiotics prophylactically. After induction of acute pancreatitis, animals received a continuous standardised intravenous volume substitution with 6 ml/kg/hour NaCl solution. A third group served as controls and received the same fluid regimen using NaCl instead of meropenem.

To evaluate bacterial and fungal infection in the long-term postoperative course, we investigated animals from the prophylactic antibiotic treatment group after seven days (n = 6). The focus of investigations in this experimental group was to evaluate the rate of fungal infection after prophylactic antibiotic treatment. Six animals underwent sham laparotomy without induction of acute pancreatitis and served as controls. Animals received intraductal saline and intravenous saline only. All parameters as described above were evaluated.

All animals were fasted for the first 24 hours of the experiment and received 6 ml/kg/hour NaCl as volume therapy. After 24 hours, animals had free access to food and water. Animals that died of technical or anaesthesiological complications were excluded from the study. In the second set of experiments, only animals that survived more than 24 hours after induction of acute pancreatitis were included.

### Bacteriology and mycology

A relaparotomy was performed 24 hours or 72 hours after induction of acute pancreatitis. First, ascites was collected and blood was drawn directly by cardial puncture after opening the thorax. Tissue of the pancreas, and the mesenteric lymph nodes of the colon and small bowel were collected under sterile conditions. Bacterial and fungal growth derived from minced tissue samples was evaluated after 72 hours of enrichment on standardised media at 37°C.

### Laboratory

Blood was drawn by intracardial puncture for measurement of amylase, lipase, and white and red blood cell count from arterial blood.

### Histological assessment

Histomorphological evaluation of the pancreas was performed by an investigator who was unaware of the experimental design using a scoring system previously described in detail [26]. In brief, the head of the pancreas was removed, fixed in 4% buffered formalin and embedded in paraffin. Coronal sections were made in the plane of the flattened pancreas and stained with H&E. Morphometric documentation included evaluation of oedema, inflammation and acinar necrosis using a scoring system from 0 (no injury) to 3 (severe injury) [26].

### Data analysis and statistics

Data is presented as mean ± standard error of the mean (SEM). Data was analysed using the SPSS Software (Version 11.5.1 for Windows, LEAD Technologies Inc., Greenwood Cliff, USA). Differences between groups were compared using analysis of variance and by the student's t-test and fisher's exact test. Statistical significance was accepted at the 5% level (p ≤ 0.05).

## Results

### Control versus prophylactic meropenem therapy (animals euthanased 24 hours after induction of acute pancreatitis)

#### Bacteriology and mycology

**Figure 2:**
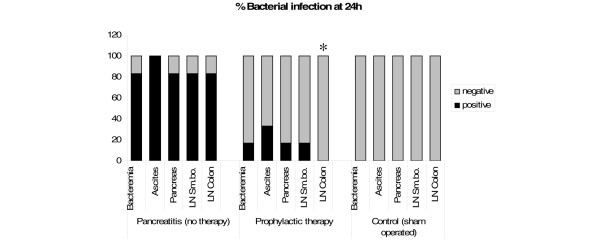
**Bacterial superinfection at 24 hours**. Meropenem given prophylactically reduced bacterial infection of blood, ascites, pancreas, and lymph nodes of the small bowel (LN Sm.bo.) and colon (LN Colon) 24 hours after induction of acute pancreatitis (*p = 0.015).

Severe bacterial infection was found in blood, ascites and pancreatic tissue of almost all animals at 24 hours after induction of acute pancreatitis (Figure 2). Germs were composed of mainly Gram-negative enteral bacteria including *Pseudomonas *and *Enterococcus*. Furthermore, bacterial specification showed typical enteral flora in mesenteric lymph nodes of the small bowel and colon. There were no significant differences between the germs in the small bowel and colonic lymph nodes.

In contrast, in the prophylactic antibiotic treatment group only one out of six rats showed bacterial infection of blood, pancreatic necrosis and lymph nodes of the small bowel. Although two animals displayed bacterial infection of ascites, mesenteric lymph nodes of the colon did not show any significant translocation (Figure 2). In addition, no fungal infection was observed at 24 hours after induction of necrotising pancreatitis.

The sham-operated animals without acute pancreatitis had no bacterial superinfection, neither in the pancreas nor in other examined tissues (Figure 2).

#### Mortality

**Figure 3:**
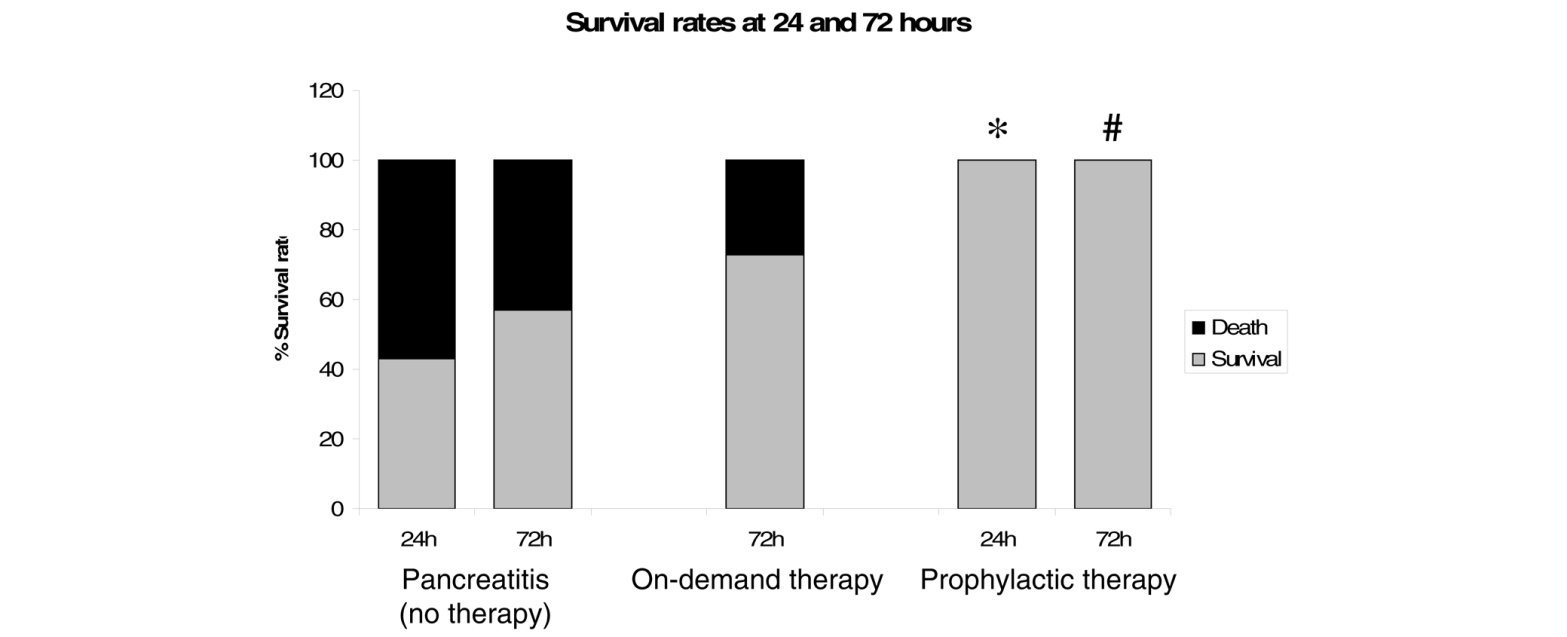
**Survival rates at 24 and 72 hours**. *p = 0.01 and ^#^p = 0.05 compared with controls.

All sham-operated control animals survived, while induction of severe necrotising pancreatitis was associated with a 24-hour mortality rate of 57.1%. In contrast, prophylactic administration of meropenem reduced mortality of acute pancreatitis significantly to 0% (p = 0.007; Figure 3). Thus, all animals that received meropenem prophylactically survived 24 hours after induction of acute necrotising pancreatitis.

#### Laboratory

In arterial blood, red and white blood cell count, amylase and lipase levels were significantly higher in the pancreatitis group compared with controls without induction of pancreatitis. However, amylase and lipase levels did not differ between early prophylactic antibiotic treatment and no treatment (data not shown).

#### Histology

Histology showed severe acute necrotising pancreatitis in all animals except shams 24 hours after induction of acute pancreatitis (Table 1). There were no significant differences between early prophylactic antibiotic treatment versus no treatment.

**Table 1:** Histological parameters at 24 hours

**Feature**	**Pancreatitis (no therapy)** Mean (range) (n = 6)	**Prophylactic therapy** Mean (range) (n = 6)	**Control (sham operated)** Mean (range) (n = 6)
Oedema	2.08 (2 to 2.5)	2.08 (2 to 2.5)	0.08 (0 to 0.5)

Inflammation	2.42 (2 to 3)	2.17 (1.5 to 2.5)	0

Necrosis	2.42 (2 to 3)	2.00 (1.5 to 2.5)	0

Histological score 0 (no injury) to 3 (severe injury) [26]

### Prophylactic versus on-demand therapy (animals euthanased 72 hours after induction of acute pancreatitis)

#### Bacteriology and mycology

**Figure 4:**
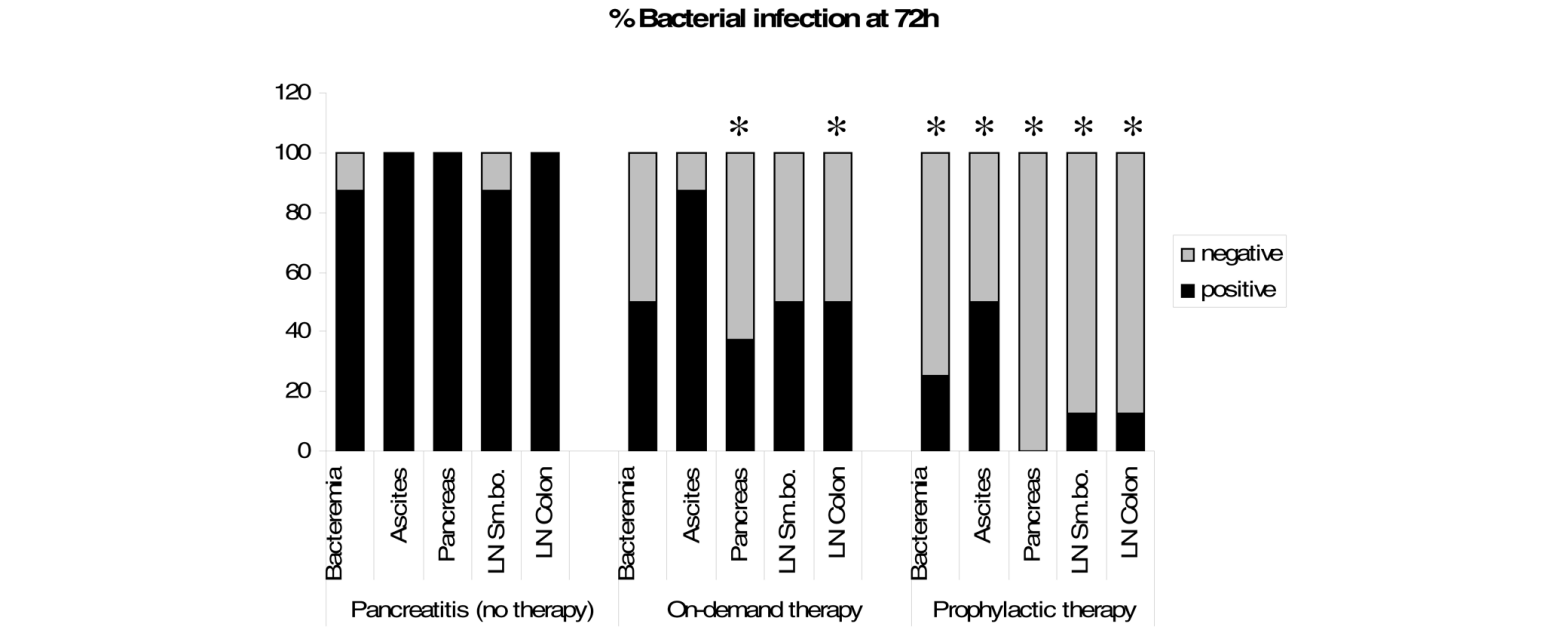
**Bacterial superinfection at 72 hours**. Both prophylactic and therapeutic antibiotic treatment reduced bacterial infection of blood, pancreatic tissue, mesenteric lymph nodes of small bowel (LN Sm.bo.) and colon (LN Colon). Compared with the control group without antibiotic treatment, prophylactic treatment reduced significantly bacterial infection of blood (p = 0.041), ascites (p = 0.039), pancreatic tissue (p < 0.001), mesenteric lymph nodes of small bowel (p = 0.010) and colon (p = 0.001). Compared with this, on-demand antibiotic treatment only reduced pancreatic superinfection and infection of mesenteric lymph nodes of the colon significantly (p = 0.026 and p = 0.039, respectively).

In controls without antibiotic treatment, severe bacterial infection of blood, ascites and mesenteric lymph nodes was found at 72 hours after induction of necrotising pancreatitis in seven out of eight cases (Figure 4). In all of these animals we found superinfection of the pancreas with enteral bacterial flora such as *Escherichia coli*, *Pseudomonas *or *Enterococcus*. Both prophylactic and therapeutic antibiotic treatment reduced bacterial infection of blood, pancreatic tissue and mesenteric lymph nodes of the small bowel and colon (Figure 4). Compared with the control group, prophylactic treatment significantly reduced bacterial infection of blood (p = 0.041), pancreatic tissue (p < 0.001) and mesenteric lymph nodes of the small bowel (p = 0.010) and colon (p = 0.001). In contrast, therapeutic antibiotic treatment on-demand only reduced pancreatic superinfection and infection of mesenteric lymph nodes of the colon compared with controls (p = 0.026 and p = 0.039). Furthermore, the decrease of bacterial infection was less pronounced compared with prophylactic antibiotic treatment. Again, no fungal infection was evident in examined tissues.

#### Mortality

Without antibiotic treatment a 72-hour mortality rate of 42.9% (6 of 14 animals) was observed in severe necrotising pancreatitis. Animals died of cardiorespiratory decompensation through the whole observation period. The time-points of death were evenly distributed without a peak. Antibiotic treatment administrated therapeutically on-demand reduced mortality to 27.3% (3 of 11 animals), while all animals (n = 8) after prophylactic antibiotic treatment survived (Figure 3). Thus, prophylactic antibiotic treatment further decreased mortality compared with therapeutic administration of meropenem. Compared with the control group without antibiotic treatment, the decrease was statistically significant (p = 0.05).

#### Laboratory

Red and white blood cell counts, amylase and lipase levels were not significantly different between the three groups (acute pancreatitis control, meropenem prophylactically and meropenem therapeutically).

#### Histology

Severe necrotising pancreatitis was evident at 72 hours after induction of acute pancreatitis. After prophylactic meropenem application, pancreatic oedema was significantly reduced compared with the control group (p < 0.05). However, there was no significant difference between the two treatment arms, therapeutic versus prophylactic meropenem administration (Table 2).

**Table 2:** Histological parameters at 72 hours

**Feature**	**Pancreatitis (no therapy)** Mean (range) (n = 8)	**On-demand therapy** Mean (range) (n = 8)	**Prophylactic therapy** Mean (range) (n = 8)
Oedema	1.63 (1.5 to 2)	1.56 (1 to 2)	1.13 (0.5 to 1.5)*

Inflammation	2.56 (2 to 3)	2.63 (2.5 to 3)	2.44 (1.5 to 3)

Necrosis	2.31 (2 to 3)	2.44 (1.5 to 3)	2.31 (1.5 to 3)

Histological score 0 (no injury) to 3 (severe injury) [26]; *p < 0.05 (prophylactic therapy versus pancreatitis)

### Long-term results (animals were euthansed seven days after induction of acute pancreatitis)

In the long-term postoperative course we focused on bacterial and fungal infection after administration of prophylactic meropenem. We did not find any fungal infection in blood, ascites, pancreas or mesenteric lymph nodes of the small bowel or colon. Furthermore, bacterial infection of ascites was only found in two out of six cases, while all other examined tissues did not show any bacterial infection. In these experiments, all animals that received prophylactic antibiotics survived the whole observation period of seven days.

## Discussion

Mortality of acute pancreatitis is about 1% to 5%, but in cases of superinfection of pancreatic necrosis mortality it increases dramatically up to 20% to 85% [2,21,24,28,29]. Due to major improvements in intensive-care management over the past decade, more patients survive the first phase of severe acute pancreatitis and therefore the prophylaxis and treatment of infected necrosis is of increasing clinical importance [30]. In many cases infected pancreatic necrosis results in the development of multiple organ failure or septic complications and is associated with high mortality rates [2,31,32]. Although various studies [8-10,13,33] and meta-analyses [15,16] have detected a beneficial role of early prophylactic treatment in acute pancreatitis, two recent double-blind studies [7,14] could not demonstrate any beneficial effects of antibiotic prophylaxis with respect to the risk of developing infected pancreatic necrosis and mortality. However, the study by Isenmann and colleagues [7] included mainly patients with mild to moderate pancreatitis, as seen by the low rate of superinfection and low mortality rate even in the control group. Regardless, the indication for antibiotic prophylaxis in necrotising pancreatitis remains controversial [3,7,17,25].

Over the past decade, it has become clear that because of variations in patient recruitment and treatment regimens it is almost impossible to include enough patients for a meaningful clinical trial [25]. Consequently, the question whether antibiotic prophylaxis in acute necrotising pancreatitis is effective in clinical practice can not be answered by clinical studies. Thus, the aim of the present study was to use a well standardised animal model to investigate the efficiency of early prophylactic antibiotic treatment compared with on-demand therapy on pancreatic infection rates, septic complications and mortality. Although, no experimental model mirrors the clinical setting completely, duct perfusion models are currently the most popular models of acute pancreatitis [34], because they are characterised by similar pathophysiological steps as the human disease [35].

In 1993, Tarpila and colleagues showed that acute pancreatitis in a rat model caused systemic bacterial colonisation [28]. They suspected bacterial translocation was the mechanism of pancreatic infection. In the present study, we found bacterial infection of blood, ascites and mesenteric lymph nodes of the colon and small bowel in almost all animals after induction of severe acute necrotising pancreatitis. The specification showed typical enteric flora in mesenteric lymph nodes of the small bowel and colon, which reflects bacterial translocation from the gut. There was no significant difference between infection of mesenteric lymph nodes of the colon and small bowel. The pathogenesis of pancreatic superinfection is not completely understood [36]. However, our findings are in agreement with the hypothesis that intra-abdominal spread by lymphatics is the pathway most likely to be involved in this process [37].

Despite the fact that in the present study a limited number of animals were available in each experimental group, we demonstrated that pancreatic and systemic infection in necrotising pancreatitis is reduced by prophylactic antibiotic treatment with meropenem (Figures 2 and 4). Furthermore, it reduced sepsis-related mortality (Figure 3). Meropenem was used because it has been shown previously that penetration into necrotic pancreatic tissue occurred in sufficient therapeutic concentrations [17,38]. Thus, meropenem might be an effective antibiotic for prevention of bacterial superinfection of necrotic pancreatic tissue and its consecutive sepsis. This is consistent with the clinical findings of many studies on acute pancreatitis [1,17,21,22] and a recent meta-analysis, which demonstrated reduced mortality and pancreatic infection rate with beta-lactam antibiotics compared with others [3].

Recent clinical experience has provided evidence that conservative management and early prophylactic antibiotic administration in sterile necrotising pancreatitis is the treatment of choice [17,21,25,31]. However, various clinical centres have diverse strategies for the treatment of acute pancreatitis [7,18,19]. To date, it remains unclear whether prophylactic antibiotic treatment or on-demand antibiotic therapy is more beneficial. Therefore, we also evaluated the effectiveness of antibiotic on-demand therapy beginning after a latency period of 24 hours after induction of necrotising pancreatitis versus early antibiotic prophylaxis (Figure 4).

Both prophylactic and therapeutic treatment on-demand reduced septic complications and mortality in experimental necrotising pancreatitis. This is supported by the results of several clinical trials performed so far [7,14,18,19]. However, our experiments showed a greater benefit for prophylactic early antibiotic treatment compared with on-demand therapy concerning superinfection of the pancreas, mortality and sepsis. Already at 24 hours, bacterial infection of lymph nodes of the colon could be significantly (p = 0.015) reduced from 83.3% to 0% by early antibiotic prophylaxis (Figure 2). These findings support the hypothesis that translocation of enteric bacteria occurs during the early stage of acute necrotising pancreatitis. This is in accordance with the results of rat experiments performed by Foitzik and colleagues [39] and Wang and colleagues [40], as well as with clinical findings published by Beger and colleagues [24] and Ammori and colleagues [41], who found endotoxaemia in 20 of 26 patients with acute pancreatitis. Therefore, as a result of our experiments we would recommend to start antibiotic therapy as early as possible after the diagnosis of pancreatic necrosis has been made. This is of particular relevance because the latest clinical trials were all statistically underpowered and could not show any difference between these two treatment modalities.

A steady rise in the incidence of pancreatic fungal infections in severe pancreatitis has been reported [42] and it was hypothesised that the widespread use of antibiotics is the cause for this phenomenon. There is also some evidence that fungal infections may worsen the outcome of acute pancreatitis, but to date this hypothesis has not been proven [43,44]. In fact, the incidence of fungal infections varies between different trials within a range of 0% to 37% [44]. In our experiments, we could not detect any fungal infection after induction of necrotising pancreatitis at any time point up to seven days. Even the use of broad-spectrum meropenem with its strong selection pressure on microorganisms did not result in detectable fungal infection. In contrast to our experimental standardised setup, patients in the clinical setting often undergo repeated interventional procedures including central venous lines or urinary catheters, which might trigger the occurrence of fungal infections during the course of necrotising pancreatitis [45]. However, with regard to the present experimental setting, we cannot exclude that fungal infections might occur at a later stage.

## Conclusion

With regard to the present study and previously published literature, meropenem is an effective antibiotic drug for the treatment of bacterial pancreatic infection in acute necrotising pancreatitis [3]. In the experimental model, both prophylactic and delayed treatment on-demand reduced septic complications and mortality. Although clinical trials have been statistically underpowered and consequently could not demonstrate any difference between antibiotic prophylaxis versus treatment on-demand, we show that in a standardised model prophylactic administration reduced pancreatic superinfection, mortality and sepsis more effectively compared with treatment on-demand. Following our experiments, the administration of antibiotics should be started earlier rather than later in the course of severe pancreatitis associated with pancreatic necrosis.

## Key messages

• The role of early antibiotic prophylaxis in severe acute pancreatitis remains controversial because clinical studies performed to date have been underpowered and results are conflicting.

• Considering the complex clinical presentation, the heterogeneous population of patients and different treatment regimens, it is difficult or near impossible to include enough patients for a meaningful clinical trial.

• In a standardised setting of experimental necrotising pancreatitis, pancreatic superinfection, bacteraemia and mortality rate were reduced significantly by early prophylactic antibiotic treatment but not delayed antibiotic treatment on-demand.

• Prophylactic antibiotic treatment is superior to treatment on-demand. Thus, antibiotics are recommended as early as possible after diagnosis of severe pancreatitis associated with pancreatic necrosis.

## Abbreviations

H & E: haematoxylin & eosin.

## Competing interests

The authors declare that they have no competing interests.

## Authors' contributions

SF, WH, RL and JW played a pivotal role in planning and carrying out the experiments. KW-S and MK performed bacterial and mycological analysis of the experimental animals. SF, WH, TH, LS, MB and JW contributed in scientific discussions as well as in preparing and writing the manuscript.
